# Alcohol does not influence trust in others or oxytocin, but increases positive affect and risk-taking: a randomized, controlled, within-subject trial

**DOI:** 10.1007/s00406-023-01676-w

**Published:** 2023-09-14

**Authors:** Leonard P. Wenger, Oliver Hamm, Christiane Mühle, Sabine Hoffmann, Iris Reinhard, Patrick Bach, Johannes Kornhuber, Georg W. Alpers, Falk Kiefer, Tagrid Leménager, Bernd Lenz

**Affiliations:** 1 Department of Addictive Behavior and Addiction Medicine, Medical Faculty Mannheim, Central Institute of Mental Health, Heidelberg University, J5, 68159 Mannheim, Germany; 2 Department of Psychiatry and Psychotherapy, Universitätsklinikum Erlangen and Friedrich-Alexander University Erlangen-Nürnberg (FAU), Erlangen, Germany; 3 Department of Biostatistics, Medical Faculty Mannheim, Central Institute of Mental Health, Heidelberg University, Mannheim, Germany; 4 Department of Psychology, School of Social Sciences, University of Mannheim, Mannheim, Germany

**Keywords:** Alcohol, Trustworthiness, Oxytocin, Positive affect, Risk-taking, Social facilitation

## Abstract

**Background:**

Alcohol consumption to facilitate social interaction is an important drinking motive. Here, we tested whether alcohol influences trust in others via modulation of oxytocin and/or androgens. We also aimed at confirming previously shown alcohol effects on positive affect and risk-taking, because of their role in facilitating social interaction.

**Methods:**

This randomized, controlled, within-subject, parallel group, alcohol-challenge experiment investigated the effects of alcohol (versus water, both mixed with orange juice) on perceived trustworthiness via salivary oxytocin (primary and secondary endpoint) as well as testosterone, dihydrotestosterone, positive affect, and risk-taking (additional endpoints). We compared 56 male participants in the alcohol condition (1.07 ± 0.18 per mille blood alcohol concentration) with 20 in the control condition.

**Results:**

The group (alcohol versus control condition) × time (before [versus during] versus after drinking) interactions were not significantly associated with perceived trustworthiness (η^2^ < 0.001) or oxytocin (η^2^ = 0.003). Bayes factors provided also substantial evidence for the absence of these effects (BF_01_ = 3.65; BF_01_ = 7.53). The group × time interactions were related to dihydrotestosterone (η^2^ = 0.018 with an increase in the control condition) as well as positive affect and risk-taking (η^2^ = 0.027 and 0.007 with increases in the alcohol condition), but not significantly to testosterone.

**Discussion:**

The results do not verify alcohol effects on perceived trustworthiness or oxytocin in male individuals. However, they indicate that alcohol (versus control) might inhibit an increase in dihydrotestosterone and confirm that alcohol amplifies positive affect and risk-taking. This provides novel mechanistic insight into social facilitation as an alcohol-drinking motive.

**Supplementary Information:**

The online version contains supplementary material available at 10.1007/s00406-023-01676-w.

## Introduction

Alcohol is among the most culturally meaningful substances that people use throughout history to induce specific bodily states [1, 2]. However, since consuming alcohol is a major health risk, it is necessary to consider relevant drinking motives [3]. An important reason for alcohol consumption is facilitation of social interaction—especially in males [4].

Alcohol may exert its socially facilitating effects by increasing perceived trustworthiness of others. Trustworthiness determines the degree of trust that is seen in other individuals [5]. Trust involves the expectation of mutually benevolent interaction and forms a central precondition for the emergence of social interactions and relationships [6–8]. Consequently, a lack of trust in the persons present hinders social interactions. An influence of alcohol consumption on perceived trustworthiness is suggested by the fact that social anxiety is both associated with an increased prevalence of alcohol use disorder (AUD) and negatively associated with trust and perceived trustworthiness regarding presented faces [9–13]. Consequently, it is assumed that socially anxious individuals consume alcohol, partly because this increases their trust in interaction partners and, as a result, facilitates social interaction. To our knowledge, there is a gap in research on whether alcohol influences perceived trust in others.

It is possible that the prosocial hormone oxytocin [14–17] mediates the hypothesized alcohol-induced increase in perceived trustworthiness, since intranasal administration of oxytocin increases perceived trustworthiness regarding presented faces as well as interpersonal trust [18, 19]. Previous studies associated oxytocin concentrations with alcohol consumption and AUD [20, 21]. In this regard, a previous study [22] identified higher oxytocin blood concentrations in patients with AUD (than in same-sex controls) at the time of hospital admission for detoxification, which decreased by the time of the follow-up survey (approximately 5 days later) and then did no longer significantly differ from those of same-sex controls. This observation may suggest that elevated oxytocin concentrations in early abstinent (defined as 24–72 h of abstinence) patients with AUD result from acute alcohol intoxication. This assumption is supported by a positive correlation between blood alcohol and oxytocin concentrations in males of this study. However apart from associative findings, the literature cannot conclusively answer the question whether alcohol consumption in an experimental setting leads to an increase in oxytocin concentration. Although some experimental studies have failed to demonstrate a significant effect of alcohol consumption on the oxytocin blood concentration [23–26], these few studies are subject to several limitations. First, the participants did not reach blood alcohol concentrations of more than about 0.9 per mille and often much less, which might have been too low to induce a significant increase in oxytocin concentration [22, 27]. Also, many previous studies did not control for food and fluid intake as well as sexual or high physical activity prior to the experiment, which may have reduced the impact of alcohol and influenced the oxytocin concentration [28–30]. Moreover, some previous studies were limited due to small sample sizes.

*Main aims of the study*: The goal of this study was to establish that in male social drinkers, an alcohol challenge (versus water; both mixed with orange juice) increases the behavioral endpoint perceived trustworthiness and that the hypothesized association between alcohol concentration and trustworthiness is mediated by salivary oxytocin concentrations. We aimed to overcome the above reported limitations of the literature. We used a male sample for several reasons: central preliminary findings such as the association between alcohol and oxytocin blood concentrations exclusively emerged in males [22]. In line with this, studies further indicate that oxytocin is more relevant to alcohol use in males than females [31, 32].

*Additional aims of the study*: In addition, administration of testosterone has been shown to reduce perceived trustworthiness of others [33, 34] and alcohol intake was demonstrated to decrease testosterone concentrations [35, 36]. However, we lack experimental data on how alcohol intake influences dihydrotestosterone (DHT) concentrations, which is a metabolite of testosterone and has a higher affinity to the androgen receptor than testosterone [37]. Therefore, we also explored the effects of alcohol on testosterone and DHT concentrations as well as a potential mediation effect regarding perceived trustworthiness. Finally, we aimed to confirm previous findings on how alcohol influences positive affect and risk-taking, since multiple studies demonstrated that alcohol administration increases positive affect and risk-taking [2, 38–41], both of which exert prosocial effects [42–48] and may, thus, be involved in alcohol-induced facilitation of social interaction. We did not examine negative affect since we did not sample depressed participants and hence, potential floor effects would have prevented the detection of an alcohol-related decrease.

## Experimental procedures

### Study description

The study was conducted at the Central Institute of Mental Health (CIMH) Mannheim, Germany. The Ethics Committee II of the Heidelberg University approved the project (ID: 2021-608) and all participants provided written informed consent and received 50 euros each for their participation. The study with its primary and secondary endpoints perceived trustworthiness and oxytocin concentrations has been preregistered in the German Clinical Trials Register (DRKS00026599). Of 79 participants who were recruited via the CIMH website and social media, 76 were analyzed after being randomized to the experimental (*n* = 56) and control conditions (*n* = 20). The randomization was based on a single sequence of random numbers. Participants in the experimental and control conditions did not significantly differ in any sociodemographic characteristic (Table 1).

**Table 1:** Sociodemographic characteristics of the study participants in the alcohol and control conditions

	Alcohol condition			Control condition			Alcohol versus control condition		
	n	M/F	SD	n	M/F	SD	t or χ^2^	*p*	
Age (years)	56	23.09	2.82	20	24.70	6.63	−1.05	0.304^§^	
Weight (kg)	56	80.29	11.53	20	85.45	12.91	−1.67	0.100^#^	
BMI (kg/m^2^)	56	23.53	2.56	20	24.74	3.32	−1.68	0.098^#^	
AUDIT score	56	9.11	3.17	20	8.80	3.90	0.32	0.754^§^	
Mass of pure alcohol applied during the experiment (g)^a^	56	93.89	10.22						
Blood alcohol concentration (per mille)									
First time point	56	0.00	0.00						
Second time point	56	0.53	0.15						
Third time point	56	1.07	0.18						
Marital status	56			20			2.87	0.339^+^	
Single	34	60.71		12	60.00				
In a relationship	22	39.29		7	35.00				
Married	0	0.00		1	5.00				
Divorced	0	0.00		0	0.00				
Other	0	0.00		0	0.00				
Educational achievement	56			20			1.22	0.785^+^	
No high school diploma^b^	0	0.00		0	0.00				
Junior high school diploma^c^	1	1.78		1	5.00				
High school diploma^d^	38	67.86		14	70.00				
Job training^e^	4	7.14		2	10.00				
University degree^f^	13	23.21		3	15.00				
Other	0	0.00		0	0.00				
Main occupation	56			20			1.24	0.832^+^	
Undergraduate	49	87.50		17	85.00				
Trainee	1	1.79		0	0.00				
Working full time	5	8.93		3	15.00				
Job-seeking	0	0.00		0	0.00				
Other	1	1.79		0	0.00				

The table shows the valid number of subjects analyzed (N), means (M) or relative frequencies (F), standard deviations (SD), and the results of ^#^t, ^§^Welch, and ^+^χ^2^ tests. AUDIT, Alcohol Use Disorders Identification Test; BMI, Body Mass Index

^a^The reported amount of pure alcohol corresponds to a total liquor mass (g) of M = 274.37 (SD = 29.86) and a total liquor volume (mL) of M = 293.39 (SD = 31.93)

^b^Kein Schulabschluss, ^c^Hauptschul- oder Realschulabschluss, ^d^(Fach)Abitur/allgemeine Hochschulreife, ^e^Lehre/Berufsausbildung, ^f^(Fach)Hochschulabschluss/Staatsexamen

Inclusion criteria were male sex, minimum age of 18 years, and being a social drinker, which was defined as regularly consuming alcohol in social contexts with blood alcohol concentrations of approximately 1.5 per mille [49]. To overcome limitations of previous studies, further criteria were defined (for an overview see Supplementary Appendix SA1). Among others, these included abstaining from drinking more than 0.5 L of fluid as well as sexual activity and high physical activity before the assessment on the day of the experiment. Also, subjects were meant to eat their last meal no later than 3 h before the start of the experiment. During a screening interview prior to the experiment, subjects were instructed to adhere to these guidelines. However, asking the subjects about their adherence on the day of the experiment revealed that only 41 participants (53.95%) had actually complied. We, thus, conducted sensitivity analyses.

**Fig. 1:**
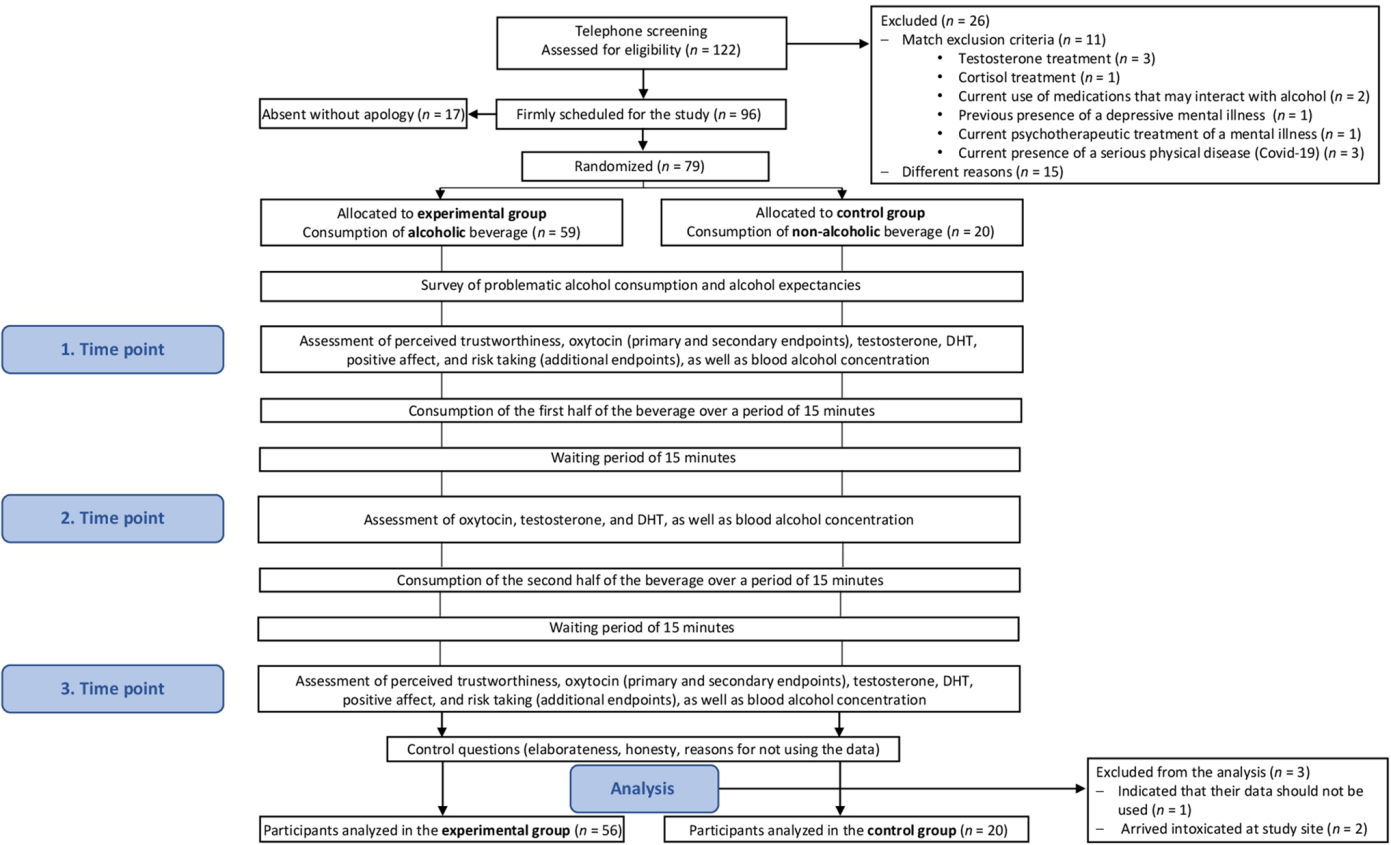
Study flow diagram

In a randomized, controlled, within-subject, parallel group, alcohol-challenge experiment, the study subjects consumed an alcohol (Vodka) orange juice mix in the alcohol condition and a water orange juice mix in the control condition (for further details see the study flow diagram in Fig. 1). The conditions were identical apart from the consumed beverage. The subjects were surveyed between 2 and 4 pm. There were three time points of measurement, with perceived trustworthiness, positive affect, and risk-taking, measured at the first and third time points, and salivary oxytocin, testosterone, DHT, as well as breath alcohol concentration measured at all three time points. The mean time span from the start of the first to the end of the third time point was M = 101.64 min. The consumed total mass of liquid was kept equal at 1200 g between the participants of the alcohol and control conditions. Between the first and second as well as the second and third time point, participants consumed 600 g of liquid each. Based on previous findings [22], the amount of vodka was calculated individually in grams to theoretically evoke blood alcohol concentrations of 1.5 per mille [50] (for details see Supplementary Appendix SA2).

### Survey of problematic alcohol consumption and alcohol expectancies

Problematic alcohol consumption was surveyed using the German version of the Alcohol Use Disorder Identification Test (AUDIT) [51]. The extent to which people expect that socially beneficial effects can be achieved through alcohol consumption was captured using a self-created measure that presented the participants with three self-descriptive statements. For each item, the subjects had to indicate on a five-point Likert-scale how much the given statement applied to them. The individual item responses were added up to a total score (for details see Supplementary Appendix SA3).

### Survey of perceived trustworthiness, positive affect, and risk-taking

The paradigm for assessing perceived trustworthiness was adopted from Theodoridou et al. [19]. The subjects were each presented with the same 30 pictures of people with neutral facial expressions in random order (see https://www.kdef.se/download-2/). For each picture, they had to indicate on a five-point Likert-scale how trustworthy they considered the person depicted. The individual item responses were added up to a total score. Positive affect was measured using the Positive and Negative Affect Schedule (PANAS) [52] and risk-taking using the Expected Involvement subscale of an adapted and translated version of the Cognitive Appraisal of Risky Events questionnaire (CARE) [53].

### Quantification of blood alcohol and salivary oxytocin, testosterone, and DHT concentrations

The blood alcohol concentration was calculated from breath alcohol content, using the AlcoTrue® M device (5040112002) by bluepoint MEDICAL (Selmsdorf, Germany). Saliva was collected using salivettes according to instructions (e.g., abstain from eating 1 h prior to saliva collection). Two saliva samples of 2 min each were collected at each of the three time points and stored at −20 °C for up to six months. After thawing, the samples were centrifuged at 1000 g for 2 min. The supernatant was used for hormone quantification. Salivary oxytocin, testosterone, and DHT concentrations were quantified using the Cayman Chemicals Oxytocin ELISA kit (500440, Cayman Chemicals, Ann Arbor, MI, USA), the Demeditec Diagnostics Testosteron frei im Speichel ELISA kit (DES6622, Demeditec Diagnostics GmbH, Kiel, Schleswig–Holstein, Germany), and the Tecan 5-alpha Dihydrotestosterone (DHT) ELISA kit (DB52021, IBL International GmbH, a Tecan Group Company, Hamburg, Hamburg, Germany), respectively, according to the manual’s instructions. For oxytocin, 50 µL were applied in parallel to a standard curve ranging from 6 to 7,500 pg/mL. Testosterone was quantified in 100 µl with a standard curve from 5 to 1,000 pg/mL. For DHT, 50 µl and a standard curve from 12.5 to 2,500 pg/mL were used. All measurements were performed blinded and within one assay run.

### Data preparation and statistical analyses

After completion of the data collection, the final dataset contained 79 cases. We removed all cases that had to be excluded due to different reasons (e.g., persons who had arrived already intoxicated or persons who indicated that their data should not be used), *N* = 76 cases (alcohol condition, *n* = 56; control condition, *n* = 20) remained in the dataset.

Alcohol-induced changes in perceived trustworthiness, positive affect, and risk-taking, as well as oxytocin, testosterone, and DHT concentrations were analyzed using two-factorial analyses of covariance (ANCOVA) with the within-subjects factor time (1 versus 3 or 1 versus 2 versus 3), the between-subjects factor group (alcohol versus control condition), and the AUDIT score as a covariate. For models with a significant group x time interaction, paired * t *tests separately for the alcohol and control conditions were calculated to compare the first and third time point. Because it was not possible to blind alcohol administration, correlations between the respective change from the first to the third time point in the alcohol condition and participants’ alcohol expectancies were calculated to control for potential expectancy effects. For models with a non-significant group x time interaction, Bayes factors were calculated to further evaluate the given absence of an effect. Structural equation modeling was used to assess the mediation hypothesis. Data were analyzed using R-Studio 2021.09.1 Build 372 (Posit PBC, Boston, MA, USA) and visualized using GraphPad Prism 8.4.3 (Graph Pad Software Inc., San Diego, CA, USA).

## Results

### Main aims: no significant alcohol-related changes in perceived trustworthiness and salivary oxytocin concentration

Means and standard deviations regarding perceived trustworthiness and oxytocin at the three time points within the experimental and control groups are displayed in Table 2.

**Table 2:** Means and standard deviations at the different time points in the alcohol and control conditions

	Time point	Alcohol condition			Control condition		
		*n*	M	SD	*n*	M	SD
Main EP							
Perceived trustworthiness	1	56	95.18	13.96	20	95.70	14.13
	3	56	92.25	17.23	20	91.80	14.29
Oxytocin concentration (in pg/mL)	1	54	682	1169	19	383	354
	2	54	1000	2662	19	934	2025
	3	54	811	1776	19	1122	1737
Additional EP							
Testosterone concentration (in pg/mL)	1	55	343	149	20	289	141
	2	55	380	220	20	340	144
	3	55	366	215	20	359	155
DHT concentration (in pg/mL)	1	55	743	289	20	699	257
	2	55	774	411	20	726	281
	3	55	728	329	20	905	502
Positive affect	1	56	3.36	0.67	20	3.14	0.60
	3	56	3.69	0.82	20	2.96	0.59
Risk-taking	1	56	89.77	18.94	20	89.10	22.11
	3	56	95.25	23.15	20	86.75	22.33

EP = endpoints; *n* = number of participants with available data; M = mean; SD = standard deviation; possible range of instruments: perceived trustworthiness (30–150), positive affect (1–5), risk-taking (30–210). For the analysis of the oxytocin concentration and the testosterone as well as DHT concentrations, three or one additional subject(s), respectively, were excluded because of missing values for at least one of the three time points

The two-factorial ANCOVA revealed a non-significant group x time interaction regarding perceived trustworthiness (F(1, 73) = 0.06, *p* = 0.803, η^2^ < 0.001) and oxytocin (F(1.57, 109.67) = 0.41, *p* = 0.616, η^2^ = 0.003). Also, Bayes factors provide substantial evidence in favor of the absence of an interaction effect for both perceived trustworthiness (BF_01_ = 3.65) and oxytocin (BF_01_ = 7.53). Besides, there was a significant main effect of time on perceived trustworthiness (F(1, 73) = 5.85, *p* = 0.018, η^2^ = 0.010), with a decrease from time point 1 to time point 3. For full model results, see Supplementary Table ST1.

A structural equation model was specified within the alcohol condition, which included the indirect effect of the blood alcohol concentration via oxytocin on perceived trustworthiness. For each variable, the difference value from the first to the third time point was used. The oxytocin model revealed no significant indirect effect of alcohol concentration via oxytocin concentration on perceived trustworthiness (z = −0.09, *p* = 0.933, β = −0.001, CI [−0.014, 0.044]).

### Additional aims

#### Group-dependent changes in salivary DHT concentration, without alcohol-related changes in salivary testosterone concentration

Means, standard deviations, and standard errors regarding salivary testosterone, and DHT concentrations at the three time points within the experimental and control groups are displayed in Table 2.

The two-factorial ANCOVA revealed no significant group x time interaction regarding testosterone (F(1.94, 139.96) = 0.65, *p* = 0.521, η^2^ = 0.002). Also, Bayes factors provide substantial evidence in favor of the absence of an interaction effect for testosterone (BF_01_ = 6.37). Regarding DHT, there was a significant group x time interaction (F(1.86, 134.01) = 5.62, *p* = 0.006, η^2^ = 0.018). Paired *t *tests showed no significant change in the alcohol condition (t(54) = −0.45, *p* = 0.658, d_z_ = −0.06), but an increase in the control condition (t(19) = 2.98, *p* = 0.008, d_z_ = 0.67). For full model results, see Supplementary Table ST1.

Structural equation models were specified within the alcohol condition, which included the indirect effect of the blood alcohol concentration via testosterone or DHT on perceived trustworthiness. For each variable, the difference value from the first to the third time point was used. The testosterone model revealed no significant indirect effects of alcohol concentration via testosterone concentration on perceived trustworthiness (z = −0.885, *p* = 0.376, β = −0.028, CI [−0106, 0.015]). Similarly, the DHT model revealed no significant indirect effect of alcohol concentration via DHT concentration on perceived trustworthiness (z = −0.830, *p* = 0.407, β = −0.035, CI [−0.156, 0.011]).

#### Alcohol-related changes in positive affect and risk-taking

Means and standard deviations regarding positive affect and risk-taking at the first and third time point within the experimental and control groups are displayed in Table 2.

**Fig. 2:**
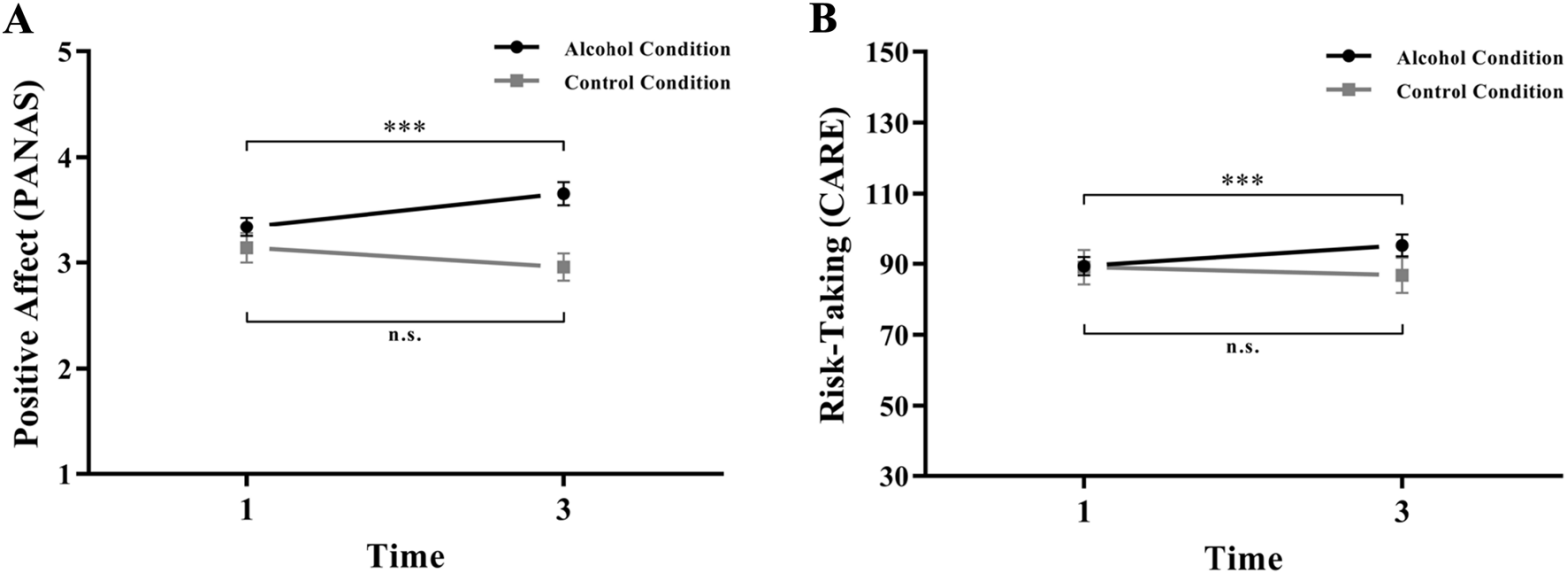
Group x time interaction on positive affect (**A**) and risk-taking (**B**). The alcohol condition versus control condition x time interactions were qualified by a significant increase in positive affect and risk-taking in the alcohol condition. PANAS, Positive and Negative Affect Schedule; CARE, Cognitive Appraisal of Risky Events questionnaire. Time 1 = prior to alcohol/water consumption (M = 0.00/M = 0.00 per mille); Time 3 = after alcohol/water consumption (M = 1.07/M = 0.00 per mille). The figure shows means and standard errors of the mean. ***p < .001

Regarding positive affect, the two-factorial ANCOVA revealed a significant group x time interaction (F(1, 73) = 10.38, *p* = 0.002, η^2^ = 0.027; Fig. 2a). Accordingly, paired *t *tests showed a significant increase in the alcohol condition (t(55) = 4.10, *p* < 0.001, d_z_ = 0.55), but not in the control condition (t(19) = −1.25, *p* = 0.226, d_z_ = −0.28). Also in terms of risk-taking, the two-factorial ANCOVA revealed a significant group x time interaction (F(1, 73) = 9.69, *p* = 0.003, η^2^ = 0.007; Fig. 2b). Accordingly, paired *t *tests showed a significant increase in the alcohol condition (t(55) = 3.83, *p* < 0.001, d_z_ = 0.51), but not in the control condition (t(19) = −1.46, *p* = 0.161, d_z_ = −0.33). For full model results, see Supplementary Table ST1.

The participants’ alcohol expectancies showed no significant correlation with the change in positive affect (t(54) = 1.39, *p* = 0.170, *r* = 0.19) or risk-taking (t(54) = −0.75, *p* = 0.455, *r* = −0.10) suggesting that there were no or only minor expectancy effects.

### Sensitivity analyses

Since several participants indicated that they had not adhered to the guidelines on the day of the experiment, all analyses were recalculated excluding these subjects. In these sensitivity analyses, the significant findings of the whole sample persisted (for details, see Supplementary Table ST2).

## Discussion

The present study examined the effects of alcohol administration on perceived trustworthiness and oxytocin concentration (primary and secondary endpoints), as well as on testosterone and DHT concentrations, positive affect, and risk-taking (additional endpoints). We were not able to verify our main hypothesis that alcohol (versus water) increases perceived trustworthiness via modulation of oxytocin. However, we detected an overall decrease in perceived trustworthiness over time in both the alcohol and the control condition. This might be explained by boredom that worsened subjects' stimulus-related attitudes after repeated presentation of the same stimuli [54]. We are also tempted to speculate that the study setting might have produced the feeling of competition and stress in the participants that could have deteriorated ratings of trustworthiness. The finding in combination with the results of the Bayesian analysis suggests that alcohol consumption does not facilitate social interaction through an increase in trust in others. There were also no indications of methodological artifacts such as a ceiling effect.

An alternate explanation for the null finding emerges by considering that alcohol has a stronger positive-euphoric effect when it occurs in naturalistic-social versus artificially isolated drinking contexts [55]. The alcohol effect on perceived trustworthiness might manifest particularly in naturalistic-social drinking contexts. In the present study, the participants consumed alcohol in an artificially isolated laboratory context. In the trustworthiness task, pictures of individuals with neutral facial expressions were presented and the participants were asked to judge without interaction. Therefore, future research is needed to translate this project in a more real-life experiment (e.g., through virtual reality [56] or ecological momentary assessments [57]).

Also, oxytocin concentrations did not significantly increase in the alcohol versus the control condition. The absence of alcohol-induced effects was supported by the results of Bayesian analyses, providing substantial evidence in favor of the lack of alcohol-related changes in oxytocin. In the present study, we addressed a population of social drinkers and quantified oxytocin in saliva samples in contrast to patients with AUD and blood serum sampling of oxytocin in the Lenz et al. investigation [22], which might explain the discrepancy concerning oxytocin. Besides, dysregulated concentrations of the soluble blood oxytocin receptor have recently been reported in patients with AUD [58]. Hence, it will be interesting to include this receptor in future studies on the effects of alcohol on the oxytocin system.

Similar to oxytocin, testosterone concentrations did not significantly change in the alcohol versus the control condition, which was again supported by the results of Bayesian analyses. For both oxytocin and testosterone, the lack of alcohol-related effects might, among others, be due to the induced alcohol concentration in the present study (M = 1.07). Regarding oxytocin, previous work suggests that alcohol-induced increases might be found at higher alcohol concentrations (largely above 1 per mille [22]), indicating that alcohol concentrations in the present study might have been too low to induce changes in oxytocin. Regarding testosterone, recent studies indicate that while low to moderate amounts of alcohol increase testosterone, high amounts of alcohol can be associated with a decrease in testosterone concentrations [59]. Given these findings, the present study might have failed to demonstrate an alcohol-induced change in testosterone because the induced alcohol concentration fell in between these opposing effects.

Interestingly, DHT did not change over time in the alcohol condition, while it increased in the control condition. We are tempted to speculate that the study setting might have produced a feeling of competition in the participants which is supported by the observed reduction of perceived trustworthiness over time and which can increase androgen concentrations [60, 61]. This study’s results suggest that potential environment effects on androgens might be inhibited by acute alcohol consumption. Future studies should implement alcohol challenges in other study settings to validate these assumptions. Also, it should be examined whether potential alcohol effects are due to alcohol-related decreases in DHT, which might be suggested by different non-experimental findings [62, 63].

This study verified our predefined hypotheses that alcohol increases positive affect and risk-taking. The results agree with the findings of McCollam et al. [40] and Lane et al. [41]. With the PANAS and CARE, the present study used alternative instruments and added external validity to the earlier findings.

## Strengths and limitations

The present study eliminated several limitations of previous studies on the effect of alcohol consumption on oxytocin concentration. We provided a larger sample size and controlled for food and water intake as well as sexual and high physical activity prior to the experiment. Even though several subjects did not adhere to the guidelines on the day of the experiment, the results persisted even when these subjects were excluded. To our knowledge, this study was the first experiment investigating the effects of a mean blood alcohol concentration above one per mille on perceived trustworthiness and oxytocin. However, the overall findings are limited to the range of blood alcohol concentrations evoked, which were between 0.6 and 1.48 (M = 1.07) per mille. Since blood alcohol concentrations were not calculated from plasma samples but inferred from breath alcohol content, the findings are also limited in this regard. Due to the specific taste of alcohol and its typical physiological effects after high doses, this study could not be blinded. However, additional analyses suggested that the findings regarding positive affect and risk-taking were not due to expectancy effects. For reasons explained earlier, the experiment was limited to males. Future studies are needed to investigate whether alcohol challenges influence perceived trustworthiness, oxytocin, testosterone, and DHT in females. The same applies to people of older age, since the current sample was rather young (M = 23.51). Also, it should be examined whether the present results transfer to plasma oxytocin measurements, since salivary oxytocin might be a weak surrogate for plasmatic oxytocin [64]. Moreover, it remains to be shown if further measures of trust in others than self-reports (e.g., economic games [18]) are also non-responsive to alcohol consumption.

## Conclusion

The present randomized, controlled, within-subject, parallel group, alcohol-challenge experiment contributes to a better understanding of how alcohol may facilitate social interaction. A blood alcohol concentration of 1.07 per mille increased positive affect and risk-taking but did not significantly influence perceived trustworthiness of others, oxytocin, or testosterone concentrations. As far as we know, this study is the first to suggest that alcohol may inhibit environmentally induced DHT increases. The results provide further insight in the role of social facilitation as an alcohol-drinking motive, which might contribute to the development of problematic alcohol use.

### Supplementary Information

Below is the link to the electronic supplementary material.

Supplementary file1 (DOCX 47 KB)

## Data Availability

Data are available upon request.
