# Supplementary material for: Alcohol does not influence trust in others or oxytocin, but increases positive affect and risk-taking: a randomized, controlled, within-subject trial
Source: Eur Arch Psychiatry Clin Neurosci. 2023 Sep 14;274(2):311–20. doi: 10.1007/s00406-023-01676-w (PMC10914917; doi:10.1007/s00406-023-01676-w)
Supplement: Supplementary file 1 — Supplementary file1 (DOCX 47 KB) [file 406_2023_1676_MOESM1_ESM.docx]

**Alcohol does not influence trust in others or oxytocin, but increases positive affect and risk taking: a randomized, controlled, within-subject trial**

*European Archives of Psychiatry and Clinical Neuroscience*

Leonard P. Wenger^*^, Oliver Hamm, Christiane Mühle, Sabine Hoffmann, Iris Reinhard, Patrick Bach, Johannes Kornhuber, Georg W. Alpers, Falk Kiefer, Tagrid Leménager, Bernd Lenz

*Corresponding author at: Department of Addictive Behavior and Addiction Medicine, Central Institute of Mental Health (CIMH), Medical Faculty Mannheim, Heidelberg University, J5, 68159 Mannheim, Germany.

E-Mail: [Leonard.Wenger@zi-mannheim.de](mailto:Leonard.Wenger@zi-mannheim.de)

Supplementary Material

[Supplementary Appendix SA1. Overview of inclusion and exclusion criteria 3](#_Toc140605429)

[Supplementary Appendix SA2. Procedure for the calculation of the liquor (vodka) quantity and overview of the performed transformations and calculation steps 4](#_Toc140605430)

[Supplementary Appendix SA3. Self-created three-item measure of alcohol expectancies 6](#_Toc140605431)

[Supplementary Table ST1. Results of analyses including the total sample 7](#_Toc140605432)

[Supplementary Table ST2. Results of sensitivity analyses after exclusion of non-compliant subjects 8](#_Toc140605433)

# **Supplementary Appendix SA1.** Overview of inclusion and exclusion criteria

*Inclusion criteria*

- Male sex
- Minimum age of 18 years (no maximum age)
- Being a ’social drinker’ (defined by regular consumption of alcohol with breath alcohol levels of at least 1.5 per mille, preferably in social contexts [48]
- Last meal eaten 3 hours before the start of the experiment
- Last cigarette smoked 3 hours before start of experiment
- On the day of the experiment, the subject refrained from sexual activity before the participation
- On the day of the experiment, the subject refrained from increased physical activity before the participation
- On the day of the experiment, the subject did not consume more than 0.5 liters of fluid before the participation
- On the day of the experiment, the subject did not expose himself to excessive stress before the participation

*Exclusion criteria*

- Current and/or previous presence of a diagnosed psychotic or depressive mental illness
- Current and/or previous presence of a diagnosed alcohol-related addictive disorder
- Current psychotherapeutic treatment of a mental illness
- Current use of medications that may interact with alcohol (e.g., antidepressants, sedatives)
- Current and/or past presence of a serious physical disease: oncological therapy during the past 10 years, metabolic disease (Addison's disease, Cushing's disease, hypogonadism, testosterone treatment), neurological disease (multiple sclerosis, Parkinson's disease, amyotrophic lateral sclerosis, stroke during the past 5 years, traumatic brain disease with persistent neurological deficit), acute cardiovascular disease (first manifestation during the past 4 weeks), myocardial infarction, decompensated heart failure, renal failure, infectious disease currently under treatment, rheumatologic disease
- Use of illicit drugs in more than 15 instances over a lifetime (cannabis excluded)
- Use of illicit drugs in the last 7 days (cocaine, opioids, amphetamines; cannabis excluded)
- Known intolerance to substances containing alcohol (alcohol sensitivity or alcohol intolerance)
- Use of steroid hormone preparations (incl. anabolic steroids) within the last 12 months

# **Supplementary Appendix SA2.** Procedure for the calculation of the liquor (vodka) quantity and overview of the performed transformations and calculation steps

The mass of pure alcohol required to evoke blood alcohol concentrations of 1.5 per mille (1.5 $\frac{g (alcohol)}{kg (body weight)}$) was calculated using the Widmark formula (BAC = $\frac{A}{p \times r}$ ). It describes the relationship between the consumed mass of pure alcohol (A; in grams), the body weight (p; in kilograms), the Widmark factor (r), and the resulting blood alcohol concentration (BAC; in g alcohol/kg body weight) [49]. To maximize congruence between estimated and actual blood alcohol concentrations, the reduction factors in the final experiment were individually calculated for all participants using the adjustment of Seidl et al. [59], which takes into account interindividual variance in body weight and height.

To calculate the individual mass of the liquor necessary to evoke a blood alcohol concentration of 1.5 per mille, a transformation of Widmark's formula was used. First, the Widmark formula, according to Gullberg [60], was transposed to A (see I). Afterwards, the calculated mass of pure alcohol (in grams) was converted into the required volume of the liquor (S; in mL), taking into account the alcohol content of the liquor (in vol.-%) as well as the specific gravity of alcohol (0.8 g/cm3) (see III). Since the present study was designed to evoke blood alcohol concentrations of at least 1.5 per mille in males and used liquor of 40% alcohol content for this purpose, the formulas could be simplified (see II and IV). Finally, the calculated volume of liquor was converted from milliliters to grams by using its density, allowing the required mass of liquor to be weighed precisely with the help of a digital balance (see V).

1. BAC = $\frac{A}{p \times r}$

***Transpose formula to A:* A = BAC** $\times$**p** $\times$**r**

1. *Simplification if the following applies:*
   - BAC = 1.5 g/kg
   - r = 0.31608 – 0.004821 $\times$ body weight (p; in kg) + 0.004632 $\times$ height (h; in centimeter) (according to Seidl et al. [57])

**A = 1.5** $\times$**p** $\times$**r = 1.5** $\times$**p** $\times$**(0.31608 – 0.004821** $\times$**p + 0.004632** $\times$**h)**

Example calculation (for a male with 70 kg body weight and 180 cm body height):

1.5 $\times$ 70 $\times$ (0.31608 – 0.004821 $\times$80 + 0.004632 $\times$180) = 83.81 g of pure alcohol

1. A = S$\times\frac{alcohol content of liquor}{100}$ $\times$ 0.8

***Transpose formula to S:* S =** $\frac{\boldsymbol{A}}{\frac{\boldsymbol{alcohol content of liquor}}{\boldsymbol{100}} \times\boldsymbol{0.8}}$

1. *Simplification if the alcohol content of liquor = 40 Vol.-%*

**S =** $\frac{\boldsymbol{A}}{\frac{\boldsymbol{40}}{\boldsymbol{100}} \times\boldsymbol{0.8}}$ **=** $\frac{\boldsymbol{A}}{\boldsymbol{0.32}}$ **=** $\frac{\boldsymbol{1}}{\boldsymbol{0.32}}\times$**A = 3.125** $\times$**A**

Example calculation: 3.125 $\times$ 83.81 = 261.91 mL of liquor

1. Density of liquor with 40% alcohol content at 20°C room temperature = 0.93518 g/ml

**S (in ml)** $\times$ **0.93518 (g/ml) = S (in g)**

Example calculation: 261.91 $\times$ 0.93518 = 244.93 g of liquor

# **Supplementary Appendix SA3.** Self-created three-item measure of Alcohol Expectancies

Instruction

German version shown to the participants: Im Folgenden möchten wir Sie um einige spezifische Angaben zu Ihrer Person bitten. Bitte geben Sie an, wie sehr jede der folgenden Aussagen im Allgemeinen auf Sie zutrifft. Zu diesem Zweck steht Ihnen eine Antwortskala mit fünf Ausprägungen zur Verfügung. Scheuen Sie sich nicht, die Skala in Ihrer ganzen Breite auszunutzen, sofern es ihrer persönlichen Einschätzung entspricht. Es gibt jedoch keine richtigen oder falschen Antworten. Wir sind allein an Ihrer persönlichen Wahrnehmung interessiert.

English Translation: Below we would like to ask you for some specific information about yourself. Please indicate how much each of the following statements applies to you in general. For this purpose, you can use a five-point response scale. Feel free to use the full range of the scale, as long as it corresponds to your personal judgment. However, there are no right or wrong answers. We are only interested in your personal perception.

Items

Original:

- Ich glaube, dass ich durch Alkoholkonsum meine sozialen Hemmungen verringern kann.
- Ich glaube, dass ich mich durch Alkoholkonsum entspannter in sozialen Kontexten verhalten kann.
- Ich glaube, dass ich durch Alkoholkonsum auf andere Personen souveräner und weniger schüchtern wirken kann.

Translation:

- I believe that I can reduce my social constraints by drinking alcohol.
- I believe that I can behave in a more relaxed way in social contexts by drinking alcohol.
- I believe that drinking alcohol makes me appear more confident and less shy towards other people.

Scale

Original:

- 1 = gar nicht
- 2 = ein bisschen
- 3 = einigermaßen
- 4 = erheblich
- 5 = äußerst

Translation:

- 1 = not at all
- 2 = slightly
- 3 = somewhat
- 4 = considerably
- 5 = extremely

# **Supplementary Table ST1.** Results of analyses including the total sample

*ANCOVA and t-test statistics including the total sample*

|  | ANCOVA | | | | | t-test (Time point 1 versus 3) | | | | |
| --- | --- | --- | --- | --- | --- | --- | --- | --- | --- | --- |
|  | Effect | F | df | *p* | η^2^ | C | t | df | *p* | d_z_ |
| **Main EP** |  |  |  |  |  |  |  |  |  |  |
| Perceived | Time | 5.85 | 1, 73 | .018 | 0.010 |  |  |  |  |  |
| trustworthiness | Group | 0.01 | 1, 73 | .930 | < 0.001 |  |  |  |  |  |
|  | Group  x time | 0.06 | 1, 73 | .803 | < 0.001 |  |  |  |  |  |
| Oxytocin  concentration | Time | 1.13 | 1.57, 109.67 | .316 | 0.009 |  |  |  |  |  |
| (in pg/mL) | Group | 0.00 | 1, 70 | .967 | < 0.001 |  |  |  |  |  |
|  | Group x time | 0.41 | 1.57, 109.67 | .616 | 0.003 |  |  |  |  |  |
|  |  |  |  |  |  |  |  |  |  |  |
| **Additional EP** |  |  |  |  |  |  |  |  |  |  |
| Testosterone concentration  (in pg/mL) | Time  Group  Group x time | 3.17  0.72  0.65 | 1.94, 139.96  1, 72  1.94, 139.96 | .047  .398  .521 | 0.011  0.008  0.002 |  |  |  |  |  |
| DHT concentration  (in pg/mL) | Time  Group | 3.19  0.11 | 1.86, 134.01  1, 72 | .048  .739 | 0.010  0.001 |  |  |  |  |  |
|  | Group x time | 5.62 | 1.86, 134.01 | .006 | 0.018 | AC  CC | -0.46  2.98 | 54  19 | 0.658  0.008 | -0.06  0.67 |
| Positive | Time | 0.71 | 1, 73 | .404 | 0.002 |  |  |  |  |  |
| affect | Group | 7.79 | 1, 73 | .007 | 0.079 |  |  |  |  |  |
|  | Group | 10.38 | 1, 73 | .002 | 0.027 | AC | 4.10 | 55 | < .001 | 0.55 |
|  | x time |  |  |  |  | CC | -1.25 | 19 | .226 | -0.28 |
| Risk-  taking | Time | 1.42 | 1, 73 | .237 | 0.001 |  |  |  |  |  |
|  | Group | 0.61 | 1, 73 | .437 | 0.008 |  |  |  |  |  |
|  | Group | 9.69 | 1, 73 | .003 | 0.007 | AC | 3.83 | 55 | < .001 | 0.51 |
|  | x time |  |  |  |  | CC | -1.46 | 19 | .160 | -0.33 |

*Note*. EP = endpoints. η^2^ = generalized eta-square. C = Condition. AC = alcohol condition, CC = control condition. The analyses were based on a sample of N = 76 subjects (AC: 56 CC: 20). For the analysis of the oxytocin concentration and the testosterone as well as DHT concentrations, three or one additional subject(s), respectively, were excluded because of missing values for at least one of the three time points.

# **Supplementary Table ST2.** Results of sensitivity analyses after exclusion of non-compliant subjects

*ANCOVA and t-test statistics after exclusion of non-adherent subjects*

|  | ANCOVA | | | | | t-test (Time point 1 versus 3) | | | | |
| --- | --- | --- | --- | --- | --- | --- | --- | --- | --- | --- |
|  | Effect | F | df | *p* | η^2^ | C | t | df | *p* | d_z_ |
| **Main EP** |  |  |  |  |  |  |  |  |  |  |
| Perceived | Time | 4.45 | 1, 38 | .042 | 0.025 |  |  |  |  |  |
| trustworthiness | Group | 0.51 | 1, 38 | .481 | 0.010 |  |  |  |  |  |
|  | Group  x time | 0.01 | 1, 38 | .924 | < 0.001 |  |  |  |  |  |
| Oxytocin  concentration | Time | 1.11 | 1.26, 44.16 | .313 | 0.020 |  |  |  |  |  |
| (in pg/mL) | Group | 0.63 | 1, 35 | .432 | 0.007 |  |  |  |  |  |
|  | Group x time | 0.70 | 1.26, 44.16 | .440 | 0.012 |  |  |  |  |  |
|  |  |  |  |  |  |  |  |  |  |  |
| **Additional EP** |  |  |  |  |  |  |  |  |  |  |
| Testosterone concentration  (in pg/mL) | Time  Group  Group x time | 1.30  0.00  1.57 | 1.94, 71.69  1, 37  1.94, 71.69 | .279  .954  .217 | 0.009  < 0.001  0.010 |  |  |  |  |  |
| DHT concentration  (in pg/mL) | Time  Group | 4.67  2.28 | 1.99, 73.54  1, 37 | .012  .140 | 0.038  0.041 |  |  |  |  |  |
|  | Group x time | 7.62 | 1.99, 73.54 | .001 | 0.060 | AC  CC | -0.62  2.74 | 30  8 | 0.538  0.026 | -0.11  0.91 |
| Positive | Time | 0.02 | 1, 38 | .895 | < 0.001 |  |  |  |  |  |
| affect | Group | 1.77 | 1, 38 | .129 | 0.034 |  |  |  |  |  |
|  | Group | 4.37 | 1, 38 | .043 | 0.028 | AC | 2.65 | 31 | .012 | 0.47 |
|  | x time |  |  |  |  | CC | -0.89 | 8 | .398 | -0.30 |
| Risk- | Time | 4.94 | 1, 38 | .032 | 0.005 |  |  |  |  |  |
| taking | Group | 0.07 | 1, 38 | .789 | 0.002 |  |  |  |  |  |
|  | Group | 8.37 | 1, 38 | .006 | 0.008 | AC | 5.25 | 31 | < .001 | 0.93 |
|  | x time |  |  |  |  | CC | -0.55 | 8 | .599 | -0.18 |

*Note*. EP = endpoints. η^2^ = generalized eta-square. C = Condition. AC = alcohol condition, CC = control condition. The analyses were based on a sample of N = 41 subjects (AC: 32 CC: 9). For the analysis of the oxytocin concentration and the testosterone as well as DHT concentrations, three or one additional subject(s), respectively, were excluded because of missing values for at least one of the three time points.
